# From Environmental Risk to Cancer Stemness: Epigenetic Regulation in Oral Squamous Cell Carcinoma

**DOI:** 10.3390/ph19030471

**Published:** 2026-03-12

**Authors:** Milica Jaksic Karisik, Katarina Zeljic, Jelena Carkic, Milos Lazarevic, Giuseppe Damante, Stefan Mitic, Jelena Milasin

**Affiliations:** 1 School of Dental Medicine, University of Belgrade, Dr. Subotica 8, 11000 Belgrade, Serbia; jelena.carkic@stomf.bg.ac.rs (J.C.); milos.lazarevic@stomf.bg.ac.rs (M.L.); 2 Faculty of Biology, University of Belgrade, 11000 Belgrade, Serbia; katarina.zeljic@bio.bg.ac.rs; 3 Department of Medical Area, University of Udine, 33100 Udine, Italy; giuseppe.damante@uniud.it; 4 Clinic for Surgery, Clinical Hospital Centre “Dragisa Misovic-Dedinje”, 11000 Belgrade, Serbia; stefan.mitic@dragisamisovic.bg.ac.rs

**Keywords:** oral squamous cell carcinoma, cancer stem cells, epigenetic modulation, targeted therapy, microRNAs, histone modifications, OSCC etiology

## Abstract

Oral squamous cell carcinoma (OSCC) represents a major global health burden and remains one of the most prevalent and aggressive malignancies of the head and neck region. Despite significant advances in surgical techniques, chemotherapy, and radiotherapy, patient outcomes have improved only modestly over recent decades. The high recurrence rate, metastatic potential, and resistance to therapy underscore the complexity of OSCC biology and the limitations of conventional treatment approaches. In recent years, the concept of cancer stem cells (CSCs) has reshaped the understanding of tumor initiation, progression, and therapeutic failure in OSCC. These cells, characterized by self-renewal capacity and phenotypic plasticity, are believed to sustain tumor growth, drive recurrence, and mediate resistance to therapy. Parallel to this, insights into epigenetic regulation, including DNA methylation, histone modifications, and non-coding RNAs, have revealed new layers of molecular heterogeneity and adaptability in oral carcinogenesis. The integration of CSC biology with epigenetic modulation offers a promising foundation for the development of targeted and personalized therapeutic strategies. Novel approaches aim to eradicate CSCs, induce their differentiation, or reprogram their malignant phenotype through the use of epigenetic inhibitors and molecular modulators. This review summarizes current knowledge on the molecular and cellular mechanisms driving OSCC pathogenesis, highlights the emerging role of CSCs and epigenetic regulators, and discusses the challenges and perspectives of translating these findings into effective clinical therapies.

## 1. Introduction

Oral squamous cell carcinoma (OSCC) is a significant global health burden and a central clinical concern in dental and maxillofacial practice. It is consistently listed among the ten most frequently diagnosed cancers worldwide; yet despite advances in biomedical research and therapeutic approaches, overall survival has improved only modestly in recent years [1,2]. OSCC is a malignant epithelial tumor arising within the oral cavity or lip, and approximately 90% of oral malignancies demonstrate a squamous cell origin [3].

According to the World Health Organization (WHO) mortality records from 2019, cancer is the leading cause of death in individuals under the age of 70 in 112 out of 183 surveyed countries (WHO Mortality Database 2000–2019) [4,5,6]. Each year, approximately 405,000 new cases of oral cancer are diagnosed worldwide, with around 66,650 of these cases reported within the European Union [7]. A particularly concerning issue is that nearly half of all oral cancer cases are detected at advanced clinical stages (III and IV), where the five-year survival rate drops below 50% [8,9].

The development of OSCC is a complex, multifactorial process that begins with the transformation of a subset of normal keratinocytes. Under the influence of various exogenous and endogenous factors, these cells gradually accumulate genetic and epigenetic alterations, which lead to dysregulated cell cycle control, impaired DNA repair mechanisms, altered differentiation patterns, and resistance to apoptosis, ultimately resulting in malignant transformation [10,11,12]. One of the prevailing views is that tumors arise from the accumulation of genetic and epigenetic changes in normal tissue stem or progenitor cells (endowed with intrinsic self-renewal and longevity), which transform them into cancer stem cells (CSCs); CSCs may also originate from differentiated cells that regain stem-like properties through epigenetic reprogramming reinforced self-renewal, plasticity, and resistance to therapy [13,14,15]. This highlights CSCs as the critical reservoir sustaining tumor progression and as a central target for epigenetically driven therapeutic strategies in OSCC [16].

## 2. Methods

A literature search was conducted to determine how environmental risk factors contribute to the development of OSCC, with a particular emphasis on the effects of epigenetic mechanisms on tumorigenesis and the stemness of tumor cells and regulation of CSC properties in OSCC. The search was conducted in the PubMed, Google Scholar, Scopus databases and included original articles published in English. A particularly detailed scoping search was performed for microRNAs in order to construct a comprehensive table of all miRNAs investigated in relation to stemness properties in OSCC. The search covered original studies published between 2009, when the first reports on this topic emerged, and 2026. The following keywords and their combinations were used: “oral squamous cell carcinoma” OR “OSCC” AND “cancer stem cells” OR “CSC” AND “microRNA” OR “miRNA” OR “miR”. Titles and abstracts were first screened for relevance, followed by full-text evaluation of eligible studies, and additional articles were identified through manual screening of reference lists. Out of 118 original articles retrieved through this search, 23 studies were selected that directly demonstrated an association between miRNA dysregulation and the stemness of cancer stem cells in OSCC.

We used a miRNA-centric network visual analytics platform, miRNet 2.0 [17] (https://www.mirnet.ca/; accessed on 26 January 2026), to functionally analyze and construct an integrative network of miRNAs (from the input list, Supplementary Materials) and their experimentally validated target genes based on miRTarBase base v9.0 [18]. Minimum network and filter nodes based on degree (all network nodes, degree cut-off 2) were used in the algorithm. To explore the Kyoto Encyclopedia of Genes and Genomes (KEGG) signaling pathways in which all target genes are significantly enriched, a hypergeometric test was performed. If the adjusted *p* value was less than 0.05, the pathway was considered significantly enriched. KEGG analysis was done within the miRNet v2.0 platform.

## 3. From Classical Theories of Carcinogenesis to Modern Concepts

Carcinomas arising from different tissues display distinct molecular features of tumor development, including variations in the activation of oncogenic signaling pathways and the patterns through which malignant cells invade and disseminate from their tissue of origin [19,20]. Nevertheless, despite this diversity, a core set of fundamental cellular and molecular processes underlies the pathogenesis of most solid tumors and forms the conceptual basis for understanding cancer biology [21].

In 2000, Hanahan and Weinberg proposed six fundamental hallmarks of cancer: the ability of tumor cells to sustain autonomous proliferation, evade growth-suppressive signals, resist programmed cell death, maintain replicative immortality, induce angiogenesis, and acquire invasive and metastatic potential [22]. A decade later, this framework was expanded to include genomic instability and tumor-promoting inflammation as enabling characteristics that accelerate the acquisition of these malignant traits [23]. Subsequent conceptual advances have further identified the reprogramming of cellular energy metabolism and evasion of immune destruction as emerging hallmarks of cancer, reflecting the dynamic adaptability of tumor cells. Importantly, these malignant capabilities do not arise in isolation. Tumors are now understood as complex tissues composed not only of cancer cells but also of various recruited and reprogrammed stromal and immune cells that collectively form the tumor microenvironment, which actively supports tumor progression. Mutations in genes involved in the organization, modulation, and maintenance of chromatin architecture are increasingly being identified and functionally linked to the regulation of gene expression and the acquisition of cancer hallmarks [24].

One of the pivotal shifts in the conceptual understanding of carcinogenesis was the realization that tumors are not uniform cell masses, but rather highly heterogeneous cellular ecosystems. Within this hierarchy, a particularly critical role is attributed to cancer stem cells, a subpopulation capable of self-renewal and driving tumor growth, recurrence, and therapeutic resistance [25,26]

Contemporary research increasingly demonstrates that cancer is not solely the outcome of genetic alterations, but that epigenetic modifications also play a crucial role in its development. These include DNA methylation, histone modifications, and gene expression regulation mediated by non-coding RNAs. Such mechanisms can lead to the upregulation of oncogenic pathways or the silencing of tumor-suppressive functions, thereby promoting molecular changes that support tumor growth and progression [27]. Environmental factors typically do not cause malignant transformation through direct genetic damage alone; they rather exert their effects by altering epigenetic regulatory networks that mediate the cellular response to external stimuli. Disruption of the epigenetic landscape may interfere with essential biological processes and consequently increase cancer susceptibility [28].

Understanding the interaction between epigenetic regulation and the genome provides important insights into cancer biology and offers potential avenues for the development of innovative diagnostic tools and targeted therapeutic strategies aimed at the prevention and treatment of malignant diseases [29,30].

## 4. Etiopathogenesis of Oral Cancer—Risk Factors and Epigenome

Present studies consistently highlight the importance of risk factors and environmental influences in the development of various cancers; however, the mechanisms through which these factors contribute to tumor initiation and progression are not yet fully understood [28]. Chronic exposure to such factors may promote carcinogenesis, tumor progression, and metastasis by inducing genetic mutations, altering epigenetic regulatory patterns, and disrupting the tumor microenvironment [31].

**Figure 1:**
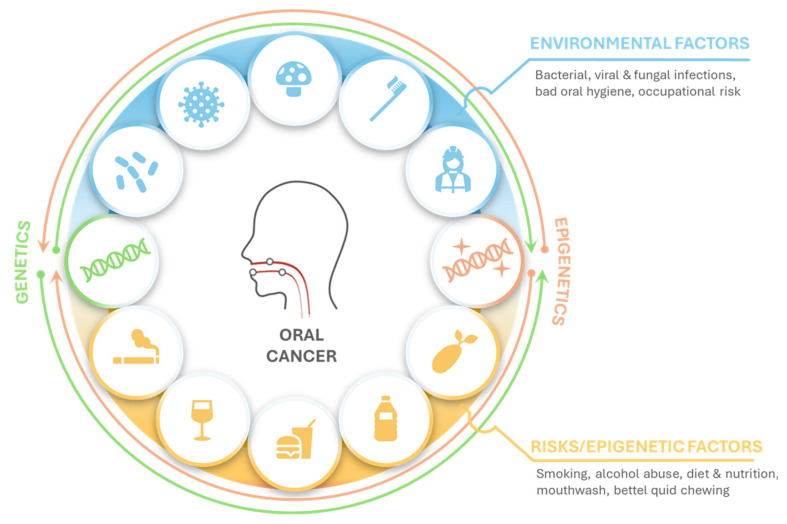
The etiology of oral cancer. Factors contributing to OSCC development. Oral cancer arises through the accumulation of genetic and epigenetic alterations. Environmental exposures (microbial infection, poor oral hygiene, occupational risk), and lifestyle-related factors (smoking, alcohol, diet, betel quid) act both directly on gene structure, and more often indirectly through epigenetic modifications, leading to dysregulated signaling, tumor initiation, and progression.

The etiology of oral cancer is therefore highly complex, resulting from the interplay of multiple environmental and behavioral risk factors that may act individually or synergistically to trigger malignant transformation (Figure 1) [32,33]. Particular attention should be given to modifiable risk factors, as lifestyle changes can significantly reduce the likelihood of developing oral cancer. These findings highlight the importance of health education, prevention strategies, and early intervention in lowering disease incidence.

Oral health status is an important risk factor for oral cancer pathogenesis. Maintaining good oral hygiene, regular tooth brushing, and routine dental check-ups can moderately reduce the risk of developing OSCC [34]. Increasing evidence highlights the role of the oral microbiome, where disruption of its balance may lead to chronic irritation and promote malignant transformation of the oral mucosa. The oral microbiome is a complex microbial community that contributes to local immune regulation and protects the host from pathogenic organisms and carcinogenic metabolites. Dysbiosis of this ecosystem can result in chronic inflammation, thereby increasing cancer risk [35]. Salivary microbiological profiling has shown that *Capnocytophaga gingivalis*, *Prevotella melaninogenica*, and *Streptococcus mitis* are significantly more abundant in patients with oral cancer compared with healthy individuals, suggesting a potential link between specific microbial signatures and OSCC development [36]. Pathogens present in the oral cavity can induce epigenetic alterations in host tissues. In head and neck squamous cell carcinoma, bacterial presence has been associated with the methylation of the *MDR1* gene, likely mediated through chronic inflammation [37]. Similarly, *H. pylori* infection can increase E-cadherin gene methylation through a mechanism in which the infection elevates IL-1β levels, activates NF-κB, and enhances DNMT activity, ultimately resulting in hypermethylation of the gene promoter region [38]. Furthermore, IL-6-induced chronic inflammation may lead to hypermethylation of tumor suppressor genes [39].

**Viral oncoproteins** can modify the activity of chromatin-remodeling complexes as well as proteins involved in miRNA processing. The E6 and E7 oncoproteins are consistently expressed in HPV-associated lesions and cancers. In addition to their canonical effects on p53 and pRb, E6 and E7 interact with a broad range of cellular proteins, including transcription factors and histone modification enzymes, leading to widespread alterations in host gene expression. Moreover, they can reshape the global transcriptional state of infected cells by influencing chromatin organization and epigenetic regulatory mechanisms [40]. The papillomavirus E2 protein binds to the C-terminal domain of Brd4, preventing the assembly of the Brd4–P–TEFb complex, which in turn allows E2 to function as a transcriptional repressor of the E6 and E7 oncogenes [41]. *DROSHA* is frequently overexpressed due to gene amplification in HPV-associated cancers, and HPV16 E6/E7 further increase the expression of *DROSHA*, leading to altered levels of DROSHA-regulated microRNAs in E6/E7–expressing cells [42].

**Tobacco** and its metabolites account for more than 80% of the risk attributable to oral cancer and influence methylation profiles in carcinoma by altering the expression of DNA methyltransferases [43]. Methylation of DNA can modulate transcription by influencing the binding of proteins that initiate and perform DNA transcription [44,45]. Cigarette smoke disrupts DNA methylation patterns by inducing DNA damage and recruiting DNMT1 to repair sites. In addition, cigarette smoke alters the activity of DNA-binding factors (e.g., *SP1*) and causes HIF-1α-dependent hypoxia that affects the availability of S-adenosylmethionine, collectively leading to persistent epigenetic changes [46]. In addition to its effects on DNA methylation, smoking also influences histone modifications. Smoking induces genotoxic and oxidative stress, which activates mitogen- and stress-activated kinase 1 (MSK1) and related signaling pathways. Activation of MSK1 leads to phospho-acetylation of histone H3 (H3S10ph/K9ac) and acetylation of histone H4 (H4K12ac) in airway epithelial cells. These modifications alter chromatin structure from a condensed to a relaxed configuration, thereby increasing transcription factor accessibility and enhancing the transcription of genes associated with inflammation, cell survival, and tumorigenesis [47]. Smoking also leads to epigenetic alterations that directly influence miRNA expression. Hypermethylation of promoter CpG islands results in the silencing of tumor-suppressor miRNAs, whereas hypomethylation can cause increased expression of oncogenic miRNAs. In addition, histone acetylation and deacetylation modify promoter accessibility, thereby regulating miRNA transcription [48]. Dysregulation of several miRNAs involved in carcinogenesis, such as miR-196a-5p, miR-10b-5p, miR-31-5p, miR-451a, and miR-144-3p, has been observed in smokers compared with non-smokers [49].

**Alcohol consumption** has been linked to epigenetic alterations, particularly changes in DNA methylation, which may contribute to cancer development. In head and neck squamous cell carcinomas, multiple studies have demonstrated that alcohol use correlates with aberrant DNA methylation patterns. Large-scale methylation profiling has shown that the degree of global DNA hypomethylation increases with alcohol exposure, suggesting that alcohol may promote genomic instability and tumor progression [50]. Smaller studies have reported that the hypermethylation of specific gene promoters, including E-cadherin (*CDH1*), *p16^INK4a^* and DAP-kinase (*DAPK1*), was significantly associated with lymph node metastasis [51]. Studies have also shown that alcohol-related dysregulation of microRNAs may contribute to carcinogenesis; for instance, alcohol-mediated overexpression of miR-30a and miR-934 in normal and HNSCC cell lines was shown to increase cell proliferation by up to two-fold and to induce the anti-apoptotic gene *BCL-2*, supporting enhanced survival of malignant cells [52].

**Figure 2:**
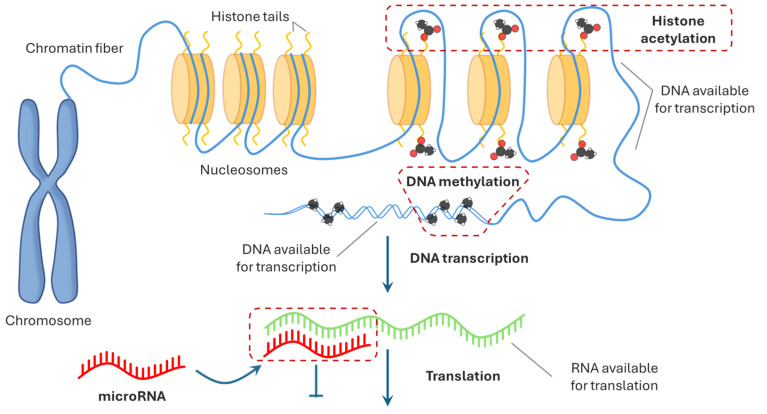
Epigenetic modifications. Epigenetic mechanisms regulating gene expression. DNA is packaged into nucleosomes formed by histone proteins, and the accessibility of chromatin determines whether genes are transcriptionally active. DNA methylation typically leads to transcriptional repression, while histone acetylation relaxes chromatin structure and promotes gene expression. Additionally, microRNAs (miRNAs) regulate gene expression post-transcriptionally by inhibiting mRNA translation or promoting mRNA degradation. Together, these epigenetic mechanisms shape the cellular phenotype without altering the underlying DNA sequence.

Taken together, these observations indicate that environmental and lifestyle risk factors can directly shape the epigenetic landscape of oral tissues, leading to altered regulation of genes involved in cell proliferation, differentiation and survival (Figure 2). By inducing changes such as DNA methylation shifts and miRNA dysregulation, these external influences may promote a cellular environment that favors malignant transformation and progression, highlighting the importance of prevention and early intervention.

## 5. The Concept of CD44^+^ Cancer Stem Cells and Their Molecular Regulation

Tumor heterogeneity is represented by the diverse phenotypic and functional characteristics of the cells within a tumor. Two main theories explain how this diversity develops. Structurally, according to the stochastic model, tumor cells are biologically similar, but their behaviors are shaped by internal and external factors, which makes tumor behavior variable and difficult to predict [53,54,55]. In contrast, the hierarchical model proposes that tumors consist of biologically distinct classes of cells with predefined functional capacities and behaviors [56,57].

A broader understanding of the CSC population shifts the perspective from a strictly “stochastic” model of clonal evolution toward a “hierarchical” model, allowing us to recognize that these two models are not mutually exclusive but are, in fact, interconnected [58,59]. Demonstrations of CSC heterogeneity and plasticity, arising from their ability to reprogram and transition between CSC and non-CSC states, further refine our understanding of CSC biology.

A major breakthrough in CSC research was the identification of surface markers that enabled the isolation and characterization of this small but biologically significant cell subpopulation. Among them, CD44 has emerged as one of the most widely studied markers. CD44 is a transmembrane glycoprotein that binds hyaluronic acid and other extracellular matrix components, and it also functions as a co-receptor for growth factors and cytokines, influencing intracellular signaling and gene expression [60,61]. Elevated CD44 expression in oral cancer correlates with poor survival, increased risk of local recurrence, radioresistance, and reduced tumor differentiation, highlighting its association with CSC-related phenotypes [54,55]. In particular, CD44 variant isoforms (CD44v) are strongly linked to self-renewal, tumor initiation, and metastatic potential, and are therefore frequently used for CSC enrichment in tumor samples and cancer cell lines [56].

The isolation of CD44^+^ CSCs is commonly performed through flow cytometry (FACS) using fluorescently labeled antibodies, or through magnetic-activated cell sorting (MACS), depending on sample type and research objectives [57]. These isolated subpopulations can then be expanded under specialized culture conditions to evaluate tumorigenicity, therapy resistance, and to develop targeted treatment strategies.

Another important CSC marker in oral squamous cell carcinoma is CD133, a transmembrane protein expressed in a very small fraction (1–2%) of tumor cells. CD133^+^ cells display increased expression of embryonic stemness markers such as OCT4 and NANOG, form tumor spheres, show higher tumorigenicity, and exhibit chemoresistance [58]. Suppression of CD133 reduces proliferation and stemness marker expression while promoting differentiation and apoptosis, confirming its role in maintaining the CSC phenotype [59].

Despite these advances in understanding cancer biology, there are still significant limitations and ongoing debates regarding the potential of CSCs as critical prognostic and therapeutic targets. Although the previously mentioned surface proteins (CD44 and CD133) have been widely used in CSC isolation, it must be noted that these markers are not exclusive to CSCs and are often expressed in normal stem cells (NSCs) and non-tumorigenic cancer cells or show a lack of correlation to cancer cell differentiation or prognosis [62,63,64,65]. For example, some researchers have even concluded that due to conflicting results of previous studies, the value of CD133 as a marker for HNSCC CSCs needs to be re-evaluated [66]. These CSC marker expression variations across tumor types highlight the importance of tissue origin and the microenvironmental context for CSC phenotypes.

Emerging evidence indicates that CSCs represent a dynamic functional state rather than a static subpopulation of cells in tumors. Recent studies have shown that stem-like features can be acquired de novo by non-CSCs through multiple context-dependent mechanisms in response to both intrinsic (genetic or epigenetic changes, and extrinsic environmental stimuli (hypoxia, inflammation, or therapeutic pressure) [67]. This broadens the origin of CSCs to not only arising from normal stem cells (NSCs) and progenitor cells with oncogenic mutations, but also hailing from terminally differentiated cancer cells that reacquire stem-like properties under various selective pressures [68]. It could also explain their abundant heterogeneity (molecular, phenotypic, metabolic and functional) not only across different tumors, but also within a single tumor. Recent advances in single-cell analyses have shown that the classical clonal model cannot fully explain intratumoral heterogeneity. In addition to genetic alterations, tumors exhibit phenotypic plasticity, enabling cancer cells to adapt to microenvironmental stress and support tumor progression, metastasis, and therapy resistance. Two forms of plasticity are recognized. Cancer cells can acquire CSC properties through both intra-lineage and trans-lineage plasticity. Intra-lineage plasticity enables dedifferentiation and reversible transitions across a spectrum of CSC states, while trans-lineage plasticity allows tumor cells to be reprogrammed into distinct cellular phenotypes and to reversibly transition between functional states, supporting stem-like and therapy-resistant characteristics [69]. In addition, the CSC ability to change (cell plasticity) has been identified as a contributing factor to their aggressive behavior and tumor progression, as well as a mediator of therapeutic resistance (8).

Another limitation of current CSC research are the variations in experimental models used across different studies. Preclinical investigation of CSCs often relies on in vitro 2D cell models which cannot accurately mimic the natural environment of cancer development, and although more advanced 3D tumor organoids and xenograft models are gaining momentum in CSC research, all long-term culture systems have significant drawbacks (accumulation of genomic and epigenetic alterations, loss of CSC subpopulations, artificial metabolic changes, lack of immune system influence) [15]. Therefore, future studies should not only aim to fine-tune these culture systems, in order to preserve cellular heterogeneity and better mimic the tumor microenvironment in vitro, but also focus on clinical trials evaluating different CSC-targeting agents. This could hopefully help bridge the gap between fundamental biological discoveries and clinical translation of CSC research in order to develop personalized CSC-targeted therapies.

**Figure 3:**
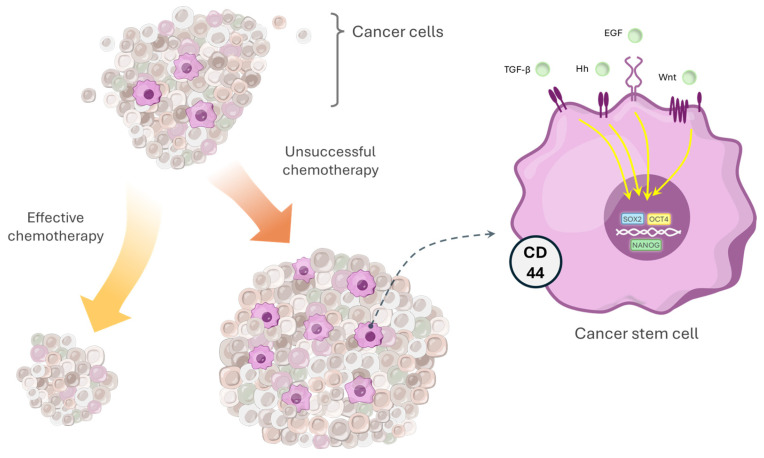
Chemoresistance in OSCC is driven by cancer stem cells (CSCs). Standard chemotherapy reduces the bulk of differentiated tumor cells but spares CSCs, identified by CD44 and stemness transcription factors (SOX2, OCT4, NANOG). The persistence of CSCs underlies recurrence and resistance, highlighting the need for therapies that specifically eliminate this subpopulation.

Cancer stem cells reside within a specialized niche that, through extracellular signaling, maintains the balance between their quiescence, self-renewal, and differentiation. The activity and self-renewal of CSCs are regulated by complex signaling networks, including microRNAs and different signaling pathways (Figure 3). It must be emphasized that although each of the numerous pathways involved in carcinogenesis has previously been studied mainly in isolation, there is extensive evidence that they seldom act independently, but are instead engaged in crosstalk, which leads to molecular events in one cascade being augmented or counterbalanced by those in another. This, in unison with other transcriptional regulators and epigenetic modifiers, can contribute to cancer development by fortifying the CSC phenotype [15], like the Wnt/β-catenin pathway, in cooperation with AKT/mTOR signaling, which collectively support the maintenance of CSCs pluripotency [70,71,72,73]. Within the Wnt/β-catenin signaling pathway, β-catenin plays a particularly important role by activating the transcription of genes such as Cyclin D1, Survivin, and c-Myc, thereby supporting the proliferation, survival, and self-renewal of CSCs [74]. In lung tumors, canonical Wnt signaling is closely associated with cancer stem cell phenotypes and has been shown to regulate the expression of OCT4, a key stemness marker involved in maintaining the CSC state [75]. More recent studies indicate that β-catenin is upregulated in the cancer stem cell subpopulation of oral squamous cell carcinoma, supporting its involvement in sustaining stemness and tumor-propagating potential [76]. Similarly, activation of the PI3K/Akt/mTOR pathway has also been shown to promote CSC maintenance; Chang et al. demonstrated that prostate cancer radioresistance is linked to EMT and enhanced CSC phenotypes via this pathway, while PI3K/mTOR signaling supports the self-renewal and tumorigenic capacity of glioblastoma stem-like cells [77,78]. Available evidence indicates that the PI3K/Akt/mTOR signaling pathway contributes to the maintenance of CSC viability, while targeting this pathway in breast cancer stem cells (CD44^+^/CD24^−^) has been associated with reduced survival and diminished self-renewal potential [79]. The JAK2/STAT3/CCND2 pathway is another pathway involved in CSC resistance which might be a promising therapeutic target, as its inhibition has been shown to enhance the efficacy of chemotherapy in colorectal cancer [80].

The Hedgehog (HH) pathway is a strictly conserved evolutionary signaling cascade involved in the regulation of the embryonic development, normal cell growth, and differentiation under physiological conditions [81]. However, aberrant activation of this pathway has been implicated in the pathogenesis of various cancers, mostly due to its role in regulating CSCs by influencing their properties, including self-renewal, differentiation, and survival [82,83]. It has been previously shown that HH expression was significantly correlated with CD133 marker expression as well as poorer survival rates in patients with OSCC [84]. The HH pathway was also found to induce EMT and activate the Wnt/ß-catenin pathway, subsequently triggering the malignant proliferation and metastasis of OSCC cells [85].

The transforming growth factor-β (TGF-β) pathway is widely known for its dual “paradoxical” role in tumor progression, since it acts both as an inhibitor and an inducer of carcinogenesis. Mainly mediated through SMAD (suppressor of mother against decapentaplegic) family proteins and their cofactors or regulators, TGF-β is produced and responded to by a wide variety of cells, resulting in a complex network with tumor-inhibiting effects in the early stages, and tumor-promoting effects in more advanced stages of carcinogenesis [86]. Previous studies have suggested that TGF-ß signaling contributes to CSC phenotype inducement in various cancers, including OSCC, as well as the existence of its close relationship with the Wnt/β-catenin and Hippo YAP/TAZ signaling pathways [87,88].

The Hippo signaling pathway, with its two downstream effectors, transcriptional coactivator PDZ binding motif (TAZ/WWTR1) and yes-associated protein (YAP), is another highly conserved signaling cascade that plays an important role in cell proliferation, tissue development, and organ size [89]. It seems to act as a bridge between EMT and CSCs, since it has been shown that the upregulation of CSC markers enhances EMT and the upregulation of EMT enhances the stemness of tumor cells in lung adenocarcinoma back, both occurring through a Hippo positive feedback loop [90]. In addition, YAP has been associated with OSCC progression, invasion, metastatic dissemination, and treatment resistance, while SOX2 was identified as a putative downstream target of TAZ in promoting CSC maintenance and tumorigenicity in HNSCC [91,92].

Transcription factors are also essential regulators of CSC identity and self-renewal. In squamous cell carcinoma, elevated SOX2 expression together with OCT4, ALDH1, and CD44 has been associated with the identification of CSC subpopulations [93]. OCT4 is known for its role in preserving totipotency in early embryonic development and pluripotency in embryonic stem cells. By maintaining cells in an undifferentiated state, OCT4 supports self-renewal, while its downregulation promotes differentiation. Its increased expression in malignancies has been linked to a more aggressive phenotype and poorer clinical outcome, including in oral cancer [94]. SOX2, which frequently partners with OCT4, reinforces this stemness network by activating genes associated with pluripotency.

NANOG is another key transcription factor that, together with OCT4 and SOX2, governs the maintenance of cellular pluripotency. NANOG expression is characteristic of undifferentiated cells, and although its precise mechanism of action remains incompletely understood, it is believed to sustain the undifferentiated state by repressing differentiation-associated genes or by enhancing the activity of other stemness regulators, such as OCT4 [95]. In cancer, NANOG is upregulated in CD133^+^ and CD44^+^ cell populations compared with their marker-negative counterparts. Through this elevated expression, NANOG supports several fundamental CSC properties, including cell proliferation, cell-cycle regulation, self-renewal capacity, epithelial–mesenchymal transition (EMT), tumorigenicity, and chemoresistance [96]. Through cooperative interactions with OCT4, NANOG and c-MYC, SOX2 contributes to the maintenance of the CSC state and has also been implicated in the reprogramming of somatic cells toward a pluripotent-like phenotype [94].

## 6. Epigenetic Regulation of CSC Signaling Networks and Its Modulation as a Therapeutic Approach

Epigenetic alterations not only initiate malignant transformation but also play a central role in maintaining the CSC phenotype. Emerging evidence suggests that, in addition to genetic traits, highly plastic epigenetic mechanisms support key features of cancer stemness, including self-renewal and stress resistance, while epigenome profiling has revealed that CSCs share epigenetic traits with embryonic stem cells, such as the repression of genes associated with cell differentiation [97].

### 6.1. DNA Methylation

DNA methylation represents one of the most extensively studied epigenetic mechanisms in oncology, as it can influence stem cell fate, while its aberrant regulation contributes to the formation of cancer stem cells, their self-renewal, metastatic potential, and the development of therapeutic resistance [98]. Methylation occurs in various regions of the mammalian genome, including promoter regions and gene bodies, through the transfer of a methyl (CH_3_) group to cytosines at the 5′ position of CpG dinucleotides; gene body CpG methylation is most commonly associated with active gene transcription in proliferating cells, whereas promoter methylation leads to gene silencing [99,100]. CSCs exhibit a distinct epigenetic landscape, where dysregulation of DNA-methylating enzymes—most notably DNMT1—drives hypermethylation and silencing of key differentiation and tumor suppressor genes such as ISL1 and FOXO3. This epigenetic repression facilitates the activation of pluripotency programs (e.g., SOX2), sustains self-renewal capacity, and enhances tumorigenic potential [101]. DNMT1-mediated methylation takes place in G0/G1, S-phase and G2/M phases of the cell cycle [102]. DNA hypermethylation of tumor-suppressor genes such as p16INK4a, PTEN, and E-cadherin removes inhibitory control over cell-cycle progression and adhesion, thereby facilitating CSC expansion and epithelial–mesenchymal transition [103]. The loss of E-cadherin can indirectly promote PI3K/Akt pathway activation by reducing PTEN expression, contributing to dysregulated survival and self-renewal signaling in tumor cells [104]. A number of genes involved in Wnt/β-catenin signaling are methylated and silenced, including the Wnt inhibitors WIF1, SFRP1–5, and DKK1, and promoter hypermethylation of these regulators leads to sustained activation of Wnt/β-catenin signaling [98,105]. Evidence from in vivo experiments showed that hypermethylation of the tumor suppressor genes HIC1 and RASSF1A confers tumor-initiating capacity to mesenchymal stem cells, supporting the role of epigenetic silencing in CSC-like reprogramming [106].

Modulating DNA methylation represents a promising therapeutic strategy in cancer treatment and several pharmacological agents have been developed to target this epigenetic dysregulation. The most widely studied are DNMT inhibitors, such as azacitidine and decitabine, which act by incorporating into DNA and trapping DNMT enzymes. This process leads to the reversal of abnormal hypermethylation and the consequent reactivation of silenced tumor suppressor genes, restoring their ability to regulate cell differentiation, apoptosis, and growth control [107].

### 6.2. Histone Modifications

Post-translational modifications of histones dynamically influence gene expression independently of alterations to the DNA sequence. Chromatin structure is regulated by histones, whose post-translational modifications—particularly acetylation and deacetylation—control DNA accessibility, with histone acetylation promoting euchromatin formation and transcriptional activation, while deacetylation leads to chromatin condensation and gene silencing [108,109,110].

Lysine acetylation is a major protein modification that regulates chromatin structure, and its dysregulation has been linked to carcinogenesis [111,112]. Studies have demonstrated that histone deacetylase (HDAC) inhibitors, such as valproic acid (VPA), enhance cellular reprogramming efficiency by preventing the removal of acetyl groups, thereby maintaining an open chromatin state that facilitates the activation of pluripotency-associated genes [113,114]. HDAC inhibitors enhance histone acetylation at promoter regions of regulatory genes such as MYC, promoting transcriptional activation and reinforcing pluripotency and stemness-associated signaling pathways [115]. One member of the important BRD subfamily, Bromodomain-containing protein 4 (BRD4), is considered an epigenetic “reader” protein and acts as a key transcriptional regulator by specifically recognizing and binding to acetylated histone H3 lysine 27 (H3K27ac) at enhancer and promoter regions of target genes, where it recruits the Mediator complex and histone-modifying enzymes to facilitate transcriptional activation [116]. BRD4 plays an important role in promoting cell cycle progression by driving cells from G0 to G1 and facilitating entry into the S phase; accordingly, BRD4 knockdown has been shown to induce cell cycle arrest, including S-phase arrest [117,118]. Therefore, BRD4 promotes stemness across multiple cancer types, and its inhibition markedly reduces cancer stem cell populations and stem cell-like properties [119].

**Figure 4:**
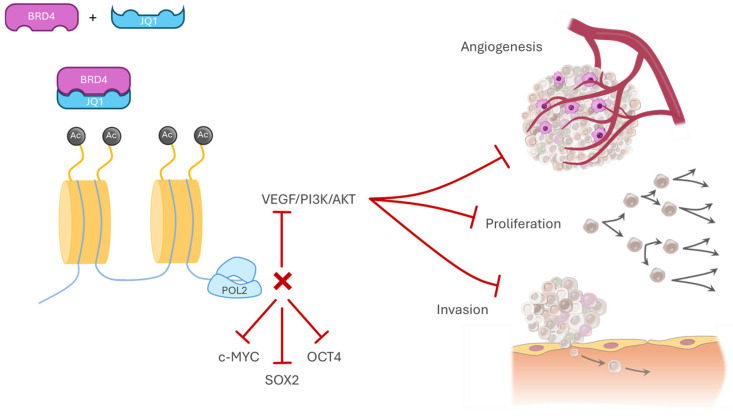
Overview of BRD4-mediated transcriptional regulation and its therapeutic targeting in cancer. BRD4 functions as an epigenetic reader that binds acetylated histones to sustain transcription of oncogenic and cancer stem cell-associated programs. Pharmacological inhibition of BRD4 by BET inhibitors such as JQ1 disrupts chromatin-associated transcriptional complexes, leading to suppression of stemness-related transcription factors and key signaling pathways involved in angiogenesis, proliferation, and invasion.

The advent of potent inhibitors of BET proteins (iBETs) represents an important step toward suppressing oncogenic networks within tumors. JQ1, an inhibitor of BRD4, interferes with the epigenetic regulation of transcription and thereby directly affects signaling networks that maintain the CSC phenotype. By inhibiting BRD4, JQ1 prevents its binding to the promoter regions of key self-renewal genes such as c-MYC, SOX2, and OCT4, leading to the suppression of stemness-associated transcriptional programs. At the same time, JQ1 inhibits the VEGF/PI3K/AKT signaling pathway, resulting in reduced proliferation, angiogenesis, and invasive capacity of CSCs (Figure 4) [120].

### 6.3. Non-Coding RNAs

Numerous findings indicate the role of non-coding RNAs, and their target genes, and signaling pathways in cancer stemness. Non-coding RNAs are key regulators of cancer drug resistance and other cancer properties: lncRNAs (longer than 200 nucleotides), circRNAs (from few hundreds to several thousands of nucleotides), and piRNAs (21–26 nucleotides), for instance, converge on a set of shared mechanisms showing the versatility of non-coding RNAs in reshaping cellular behavior. All three classes participate in gene expression regulation, i.e., its fine-tuning, often by acting as molecular sponges for microRNAs or by binding RNA-binding proteins to alter transcriptional and post-transcriptional processes. They also contribute to epigenetic remodeling, guiding chromatin modifiers or silencing elements that control oncogene and tumor suppressor activity, and intersect with different cell signaling pathways [121]. However, microRNAs (miRNAs) remain the most extensively studied ncRNAs due to their central role in controlling gene networks that drive chemoresistance, tumor progression, and cancer stem cell maintenance [122,123].

**Figure 5:**
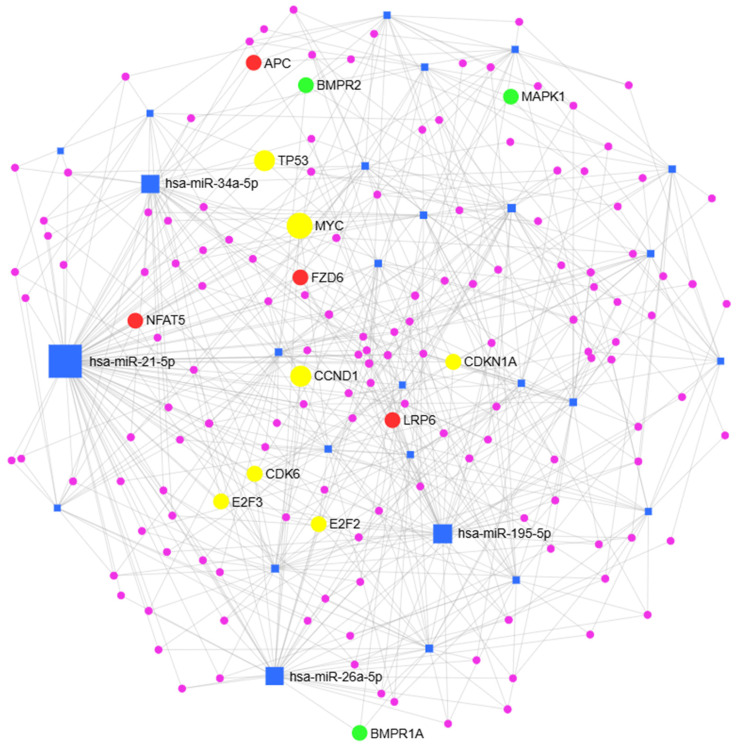
Stemness-associated miRNAs—target gene interaction network. Blue squares represent miRNAs, and circles represent experimentally validated genes regulated by miRNAs according to miRTarBase v9.0. Hub miRNAs are labeled, and their size depends on the centrality measures: degree (number of miRNA–target gene interactions) and betweenness (number of shortest paths going through the node). Red circles represent genes significantly enriched in the Wnt signaling pathway, green circles represent genes significantly enriched in the TGF-ẞ signaling pathway, and yellow circles represent genes enriched in the cell cycle. The size of the circles is proportional to the number of significantly enriched pathways in which a specific gene is localized. Even though highlighted in yellow to indicate the cell cycle, TP53 and CCND1 were also enriched in the Wnt signaling pathway, while MYC was enriched in both the Wnt and the TGF-β signaling pathways. Purple circles represent target genes enriched in all other pathways.

A list of miRNAs associated with CSCs was obtained from a previously published review paper (Table S1) [124]. This list included miRNAs supported by experimental evidence across various cancer types, including OSCC. For the purpose of this review, we aimed to investigate whether miRNAs regulate genes within signaling pathways critical for stemness, while avoiding redundancy by not reiterating previously reported miRNA targets (reviewed by Yoshida et al.) [124]. Thus, we performed an exploratory in silico analysis to provide an integrative perspective on stemness associated miRNAs independent of specific cancer context. Stemness-associated miRNAs and their target genes form a complex regulatory network of interaction (Figure 5). Four miRNAs, namely miR-21-5p, miR-195-5p, miR-34a-5p and miR-26a-5p, were identified as the central hubs of the network based on the highest centrality measured by degree and betweenness parameters. Among the significant signaling pathways, there were those of importance to cancer stemness (Table S1). Key stemness signaling pathways, including Wnt, TGF-ẞ and the cell cycle, were identified. This in silico exploration reflects the miRNA regulatory complexity underlying cancer stemness and supports the importance of CSC regulation by miRNAs. It might also serve as a starting point for future functional studies of miRNAs in the regulation of CSCs in OSCC, and other cancer types.

There are numerous pieces of evidence that miRNAs play a pivotal role in gene expression regulation and substantially contribute to the initiation and maintenance of cancer stem cells by modulating stemness-associated markers and key signaling pathways [125]. In addition to directly affecting the transcription of pluripotency-associated genes, a single miRNA can simultaneously regulate multiple stemness-related markers; for instance, miR-145 directly targets NANOG, OCT4, SOX2 in both embryonic stem cells and CSCs, thereby suppressing their self-renewal capacity [123]. In addition, numerous miRNAs modulate CSC phenotypes by regulating key developmental signaling pathways, including Wnt/β-catenin, PI3K/Akt, and NF-κB, which are essential for maintaining stemness, survival, and therapy resistance [76,126,127]. Upregulation of the tumor suppressor miR-34a in OSCC attenuates cancer stem cell-like characteristics through the repression of CSC-associated transcription factors, including SOX2, NANOG, and OCT3/4 [128]. Similarly, in prostate cancer, miR-143 and miR-145 have been implicated in the progression of bone metastases by modulating cancer stem cell-associated characteristics [129]. In liver cancer, miR-155 promotes cancer stem cell-like properties through NF-κB-dependent upregulation, correlating with increased expression of CSC markers (CD90, EpCAM, OCT4) and enhanced spheroid formation, while its inhibition suppresses stemness by downregulating SOX2 and OCT4 [130]. MiR-21, a well-known oncomiR, is frequently overexpressed in oral cancer, where it represents a poor prognostic biomarker and is associated with tumor growth, invasion, and metastasis, as it contributes to the maintenance of CSC stemness. Its inhibition leads to a substantial downregulation of SOX2, OCT4, Nanog and Wnt/β-catenin signaling pathway [76]. In this context, epigenetic therapies such as BRD4 inhibition offer a promising strategy to disrupt CSC transcriptional programs. Moreover, combining JQ1 with miR-21 inhibition may produce a synergistic effect, enhancing the activation of caspase-3 in CSCs and thus promoting apoptosis [131]. Refer to Table 1.

## 7. Therapeutic Strategies Targeting CSC-Associated Pathways and Epigenetics in OSCC

Although substantial progress has been made in elucidating the epigenetic and stemness-associated mechanisms underlying OSCC progression, their clinical translation remains partial. Nevertheless, several approved drugs and new agents directly or indirectly target molecular pathways discussed in this review, providing a rationale for CSC- and epigenetically guided therapeutic strategies (Table 2).

### 7.1. Approved Therapies Targeting CSC-Related Signaling Networks and Epigenetic Regulation

Cetuximab (erbitux), an EGFR-inhibiting monoclonal antibody, is currently approved by both the Food and Drug Administration (FDA) and the European Medicines Agency (EMA) for the treatment of head and neck squamous cell carcinoma, including OSCC [150]. As EGFR activation converges on PI3K/Akt/mTOR and STAT3 signaling—pathways critically involved in CSC survival and maintenance—cetuximab may exert partial anti-CSC effects through disruption of stemness-supportive signaling networks [151]. However, intrinsic and acquired resistance remain major clinical limitations, potentially due to the persistence of CSC populations. Pembrolizumab and nivolumab are FDA-approved drugs for recurrent and metastatic HNSCC [152]. Evidence suggests that CSCs contribute to immune evasion by upregulating PD-L1 expression and activating STAT3-dependent immunosuppressive pathways [153,154]. Thus, immune checkpoint blockade may indirectly target CSC-mediated immune resistance.

Although no epigenetic drug is currently approved specifically for OSCC, several agents targeting DNA methylation (DNA methyltransferase (DNMT) inhibitors) and histone modification (histone deacetylase (HDAC) inhibitors) have demonstrated clinical efficacy in hematologic malignancies [155,156].

Azacitidine and decitabine (DNMT Inhibitors) are FDA approved drugs for myelodysplastic syndromes and acute myeloid leukemia [157,158]. These nucleoside analogs incorporate into DNA and block DNMT enzymes, leading to passive demethylation and reactivation of silenced tumor suppressor genes. Given the role of DNMT1-mediated hypermethylation in OSCC—particularly in silencing the PI3K-AKT signaling pathway—DNMT inhibition may disrupt CSC maintenance [159]. Preclinical evidence in head and neck cancer models suggests that DNMT inhibitors may also sensitize tumors to chemotherapy and radiotherapy [160].

Vorinostat and romidepsin, two HDAC inhibitors, have been approved by the FDA for cutaneous T-cell lymphoma [161]. HDAC inhibition increases histone acetylation, relaxes chromatin and promotes transcriptional reactivation of suppressed genes [162]. In solid tumors, including HNSCC models, HDAC inhibitors have been shown to: reduce CSC-like phenotypes, impair tumor sphere formation, and enhance sensitivity to conventional therapies [163,164,165]. However, clinical efficacy in head and neck cancer remains under investigation, and combination regimens are likely required to achieve durable responses.

### 7.2. Emerging Agents Targeting CSC-Related Signaling Networks and Epigenetic Regulation

Bromodomain and extraterminal (BET) proteins, particularly BRD4, represent central epigenetic “readers” sustaining oncogenic transcriptional programs [166]. JQ1, a preclinical BET inhibitor, has demonstrated the ability to suppress *cMYC*, *SOX2*, and *OCT4* transcription, disrupt VEGF/PI3K/AKT signaling, and reduce CSC populations in multiple tumor models [167,168,169]. Although JQ1 itself is not clinically approved, several BET inhibitors have entered early-phase clinical trials in solid and hematologic malignancies [170]. Given BRD4’s role in maintaining stemness-associated transcriptional networks in OSCC, BET inhibition represents a highly promising CSC-directed strategy that warrants further clinical exploration.

Multiple inhibitors targeting PI3K/Akt/mTOR and JAK/STAT signaling pathways are approved in other cancer types and may have translational relevance for OSCC. Examples include: everolimus (mTOR inhibitor), alpelisib (PI3Kα inhibitor), and ruxolitinib (JAK1/2 inhibitor) [171,172,173]. These agents could interfere with signaling cascades essential for CSC survival, proliferation, and therapy resistance. Although clinical results in HNSCC have been modest, rational combination strategies integrating pathway inhibitors with epigenetic modulators or immunotherapy may improve CSC targeting.

MRX34, a liposomal miR-34a mimic, entered early-phase clinical trials in solid tumors, marking one of the first attempts at miRNA replacement therapy in oncology [174]. Although development was halted due to immune-related adverse events, the trial demonstrated the clinical feasibility of miRNA-based strategies.

Given the strong involvement of miR-21, miR-34a, miR-145, and other miRNAs in regulating OSCC stemness, targeted miRNA mimics or anti-miRNA oligonucleotides may offer future CSC-directed therapeutic options, particularly in combination with epigenetic inhibitors.

## 8. Conclusions

Oral squamous cell carcinoma remains a major clinical challenge due to its pronounced heterogeneity, tendency for recurrence, and development of therapeutic resistance. The accumulation of epigenetic alterations within the CSC population represents a central mechanism that maintains their self-renewal capacity, plasticity, and resistance to treatment. Environmental and lifestyle risk factors, including tobacco use, alcohol consumption, microbial dysbiosis, and viral infections, contribute to OSCC development largely through epigenetic reprogramming. Understanding the interplay between key signaling pathways (Wnt/β-catenin, PI3K/Akt/mTOR, JAK/STAT) and epigenetic regulation provides opportunities for the design of targeted and epigenetically guided therapeutic strategies. The integration of these insights into clinical practice represents a critical direction for future advancements in OSCC management, thereby paving the way for the identification of novel targeted therapy modalities.

## 9. Future Directions

Future research should focus on translating advances in cancer stem cell biology and epigenetic regulation into clinical practice for improved OSCC management. The identification of reliable epigenetic biomarkers for early detection, prognosis, and prediction of therapeutic response may support more precise treatment decisions. Targeting DNA methylation, histone modifications, and non-coding RNA networks that maintain CSC survival and resistance represents a promising therapeutic direction. In addition, combination strategies that integrate epigenetic modulators with targeted therapies and immunotherapy, supported by emerging technologies such as single-cell analysis and liquid biopsy, may improve monitoring of tumor heterogeneity and guide personalized treatment. Well-designed translational and clinical studies remain essential for the development of precision medicine approaches aimed at eliminating CSCs and improving long-term patient outcomes.

## Figures and Tables

**Table 1:** MicroRNAs involved in the regulation of cancer stem cell properties in oral squamous cell carcinoma.

miRNA	Target Gene(s)	Signaling Pathway	Effect on CSC Phenotype	Experimental Model	References
miR-200b/miR-200c	*ZEB1*, *BMI1*	EMT/Wnt-β-catenin	Regulates epithelial phenotype, loss promotes EMT, stemness and chemoresistance	In vitro, tissue samples	[132,133]
miR-203	*BMI1*	Stemness/Notch	Suppression induces CSC expansion and EMT in HPV-related OSCC	In vitro/in vivo SCC-25, HSC-3, HSC-4, SAS, HOK-16B (cell lines)	[132,134]
miR-138	*RHOC, EZH2*	EMT/Epigenetic	Loss enhances invasion and CSC traits	In vitro, tissue samples	[132]
miR-145	OCT4 (encoded by *POU5F1*), *SOX2*, CD44	Pluripotency network	Inhibits self-renewal and tumorigenicity of OSCC CSC	In vitro, FaDu, KB, HGF1, and HEK 293T	[135,136]
miR-205	*ZEB1*	EMT	Maintains epithelial state, loss favors CSC phenotype	In vitro, tissue samples	[132]
miR-204	*SOX4,* Slug *(SNAI2)*	TGF-β/EMT	Suppresses stemness and EMT in OSCC CSC	In vitro/in vivo SAS, OECM-1	[137]
miR-218	*BMI1*	Wnt/β-catenin	Inhibits CSC maintenance and tumor growth	In vitro, Primary cell cultures	[138]
miR-15b	*TRIM14*	PI3K/AKT	Reduces stemness and chemoresistance	In vitro, SCC-25	[139]
miR-143	*CD44*	CSC niche/adhesion	Suppresses proliferation and angiogenesis	In vitro, KB, HGF1, and HEK 293T	[136]
miR-590	VE-cadherin (*CDH5)*	Angiogenesis	Promotes vascularization and CSC support	In vitro, KB, HGF1, and HEK 293T	[136]
miR-495	*HOXC6*	TGF-β	Inhibits EMT, invasion and CSC self-renewal	In vitro, tissue samples	[140]
miR-134	*LAMC2*	PI3K/AKT	Suppresses invasion and survival of CSC	In vivo/in vitro	[141]
miR-146a	*CD24, NUMB*	AKT/Notch/β-catenin	Maintains CSC self-renewal and plasticity	In vitro/in vivo, CAL-27, SAS, SCC-25, HN5	[142,143]
miR-224-5p	*PANX1*	Apoptosis	Promotes chemoresistance of CD44high CSC	In vitro, HSC-3	[144]
miR-29a	*MCL-1*	PI3K/AKT/Apoptosis	Enhances survival and stemness via anti-apoptotic signaling	In vitro, SAS, CAL-27	[145]
miR-485-5p	*KRT17*	β-catenin/FAK/Src	Suppresses stemness and chemoresistance	In vitro/in vivo, SAS, CAL-27	[146]
miR-509	*PLK1*	Cell cycle	Inhibits CSC proliferation and self-renewal	In vitro, SAS, OECM-1	[147]
miR-21	*BCL-2*, *CASP3*	Wnt/β-catenin	Promotes CSC survival, stemness and therapy resistance	In vitro, Primary cell cultures, SCC-25	[76]
miR-204	SOX2, OCT4, ALDH1	HMGB1	lncRNA-mediated activation of CSC phenotype	In vitro, SAS, CAL-27	[148]
miR-376a	NRP1, ALDH1	Migration/Angiogenesis	Suppresses CSC invasion and self-renewal	In vitro, SAS, OECM-1	[149]
miR-143	CD44, SOX2	CSC maintenance	lncRNA-driven enhancement of stemness	In vitro, SAS, CAL-27	[145]

**Table 2:** Therapeutic strategies targeting CSC-associated pathways and epigenetic regulation in cancers.

Target Molecule/Pathway	Drug	Approval Status	Cancer Indication	Relevance to OSCC CSC Biology
EGFR	Cetuximab	FDA/EMA approved	HNSCC*	Indirect inhibition of PI3K/Akt and STAT3 signaling; may reduce CSC survival
PD-1	Pembrolizumab	FDA/EMA approved	Recurrent/metastatic HNSCC	Modulates CSC-mediated immune evasion
PD-1	Nivolumab	FDA/EMA approved	Recurrent/metastatic HNSCC	Counteracts immune suppression linked to CSC plasticity
DNMT	Azacitidine	FDA/EMA approved	MDS*, AML*	Reverses hypermethylation of tumor suppressors; may disrupt CSC maintenance
DNMT	Decitabine	FDA/EMA approved	MDS, AML	Epigenetic reprogramming; potential CSC differentiation induction
HDAC	Vorinostat	FDA approved	CTCL*	Reduces CSC-like phenotype; enhances therapy sensitivity
HDAC	Romidepsin	FDA approved	CTCL	Chromatin remodeling; experimental use in solid tumors
BRD4 (BET)	JQ1	Preclinical	N.A.	Suppresses MYC, SOX2, OCT4; reduces CSC transcriptional programs
mTOR	Everolimus	FDA/EMA approved	Multiple cancers	Targets PI3K/Akt/mTOR signaling sustaining CSC survival
PI3Kα	Alpelisib	FDA approved	Breast cancer	Potential CSC-targeting via PI3K inhibition
JAK1/2	Ruxolitinib	FDA approved	Myelofibrosis	Disrupts JAK/STAT-driven CSC resistance
miR-34a	MRX34	Phase I (terminated)	Solid tumors	Proof-of-concept for miRNA-based CSC modulation

* Head and neck squamous cell carcinoma (HNSCC), Myelodysplastic syndromes (MDS), Acute myeloid leukemia (AML), Cutaneous T-cell lymphoma (CTCL).

## Data Availability

No new data were created or analyzed in this study. Data sharing is not applicable to this article.

## Supplementary Materials

The following supporting information can be downloaded at https://www.mdpi.com/article/10.3390/ph19030471/s1, Table S1. KEGG pathways enriched in genes targeted by stemness associated miRNAs. List of stemness associated miRNAs used as an input for in silico exploratory analysis of interactions, retrieved from https://pubmed.ncbi.nlm.nih.gov/33598508/ (accessed on 26 January 2026).
