# Supplementary material for: From Environmental Risk to Cancer Stemness: Epigenetic Regulation in Oral Squamous Cell Carcinoma
Source: Pharmaceuticals (Basel). 2026 Mar 12;19(3):471. doi: 10.3390/ph19030471 (PMC13029695; doi:10.3390/ph19030471)
Supplement: Supplementary file 1 [file pharmaceuticals-19-00471-s001.zip › pharmaceuticals-4148699-supplementary.pdf]

Supplementary Materials

List of stemness associated miRNAs used as an imput for *in silico* exploratory analysis of interactions, retreived from https://pubmed.ncbi.nlm.nih.gov/33598508/

hsa-let-7, hsa-miR-106b, hsa-miR-10b, hsa-miR-1181, hsa-miR-1275, hsa-miR-128-3p, hsa-miR-135a, hsa-miR-135b, hsa-miR-136, hsa-miR-142a-3p, hsa-miR-145, hsa-miR-146, hsa-miR-181b, hsa-miR-194-5p, hsa-miR-195-5p, hsa-miR-1976, hsa-miR-200, hsa-miR-200b-3p, hsa-miR-200c, hsa-miR-205, hsa-miR-21, hsa-miR-214-3p, hsa-miR-21-5p, hsa-miR-22, hsa-miR-22-3p, hsa-miR-22-5p, hsa-miR-26a-5p, hsa-miR-28-5p, hsa-miR-302c, hsa-miR-30b, hsa-miR-320, hsa-miR-340, hsa-miR-34a, hsa-miR-486-5p, hsa-miR-600, hsa-miR-603, hsa-miR-7, hsa-miR-7-5p, hsa-miR-873, hsa-miR-92a

Supplementary Table S1. KEGG pathways enriched in genes targeted by stemness associated miRNAs

Name	Hits	P	Adjusted P
Pathways in cancer	27/310	3.46e-17	3.98e-15
Prostate cancer	16/87	2.83e-15	1.63e-13
Glioma	13/65	5.04e-13	1.93e-11
Melanoma	12/68	2.12e-11	6.09e-10
Chronic myeloid leukemia	12/73	5.11e-11	1.18e-9
Bladder cancer	8/29	1.37e-9	2.63e-8
p53 signaling pathway	10/68	7.6e-9	1.25e-7
Pancreatic cancer	10/69	8.81e-9	1.27e-7
Non-small cell lung cancer	9/52	1.03e-8	1.32e-7
Small cell lung cancer	10/80	3.83e-8	4.4e-7
Focal adhesion	14/200	1.05e-7	0.0000011
Endometrial cancer	7/44	9.37e-7	0.00000898
ErbB signaling pathway	9/87	0.00000103	0.00000911
HTLV-I infection	12/199	0.00000482	0.0000396
Epstein-Barr virus infection	8/91	0.0000148	0.000113
mTOR signaling pathway	6/45	0.0000172	0.000124
Colorectal cancer	6/49	0.0000285	0.000193
Acute myeloid leukemia	6/57	0.0000683	0.000436
Renal cell carcinoma	6/60	0.0000915	0.000554
Toxoplasmosis	7/93	0.000143	0.000822
Jak-STAT signaling pathway	7/99	0.000212	0.00116
Hepatitis C	7/100	0.000226	0.00118
Measles	7/102	0.000256	0.00128
Thyroid cancer	4/28	0.000372	0.00176
MAPK signaling pathway	11/265	0.000383	0.00176
Apoptosis	6/83	0.000552	0.00244
Neurotrophin signaling pathway	7/123	0.000801	0.00329
Chagas disease (American trypanosomiasis)	6/89	0.000801	0.00329
Cell cycle	7/124	0.00084	0.00333

Toll-like receptor signaling pathway	6/97	0.00126	0.00467
Fc gamma R-mediated phagocytosis	6/97	0.00126	0.00467
Adherens junction	5/70	0.00173	0.00622
Regulation of actin cytoskeleton	8/182	0.00182	0.00634
Wnt signaling pathway	7/144	0.00202	0.00683
Progesterone-mediated oocyte maturation	5/80	0.00313	0.0103
Viral myocarditis	3/26	0.00403	0.0129
Bacterial invasion of epithelial cells	4/56	0.00514	0.016
Cholinergic synapse	5/95	0.00654	0.0198
Insulin signaling pathway	6/137	0.00711	0.021
Chemokine signaling pathway	7/189	0.00903	0.026
Dorso-ventral axis formation	2/12	0.00951	0.0267
Influenza A	5/107	0.0107	0.0293
B cell receptor signaling pathway	4/75	0.0142	0.0371
Fc epsilon RI signaling pathway	4/75	0.0142	0.0371
VEGF signaling pathway	4/76	0.0149	0.0381
Osteoclast differentiation	5/119	0.0164	0.041
TGF-beta signaling pathway	4/84	0.0208	0.0502
Carbohydrate digestion and absorption	2/18	0.021	0.0502
Tuberculosis	6/174	0.0214	0.0502
NOD-like receptor signaling pathway	3/49	0.0233	0.0536
Natural killer cell mediated cytotoxicity	5/138	0.029	0.0654
T cell receptor signaling pathway	4/98	0.0342	0.0756
African trypanosomiasis	2/25	0.0389	0.0844

KEGG - Kyoto Encyclopedia of Genes and Genomes; Hits - total number of genes in pathway / number of genes in miRNA-gene interaction network involved in the pathway; p – p values obtained by hypergeometric test; adjusted p value for false discovery rate (FDR)
